# Morphological and Ultrastructural Features of Formation of the Skin of Wheat (*Triticum aestivum* L.) Kernel

**DOI:** 10.3390/plants10112538

**Published:** 2021-11-21

**Authors:** Inna A. Chaban, Alexander A. Gulevich, Elena A. Smirnova, Ekaterina N. Baranova

**Affiliations:** 1 Plant Cell Biology Laboratory, All-Russia Research Institute of Agricultural Biotechnology, Timiryzevskaya 42, 127550 Moscow, Russia; kinggobi@yandex.ru; 2 Plant Cell Engineering Laboratory, All-Russia Research Institute of Agricultural Biotechnology, Timiryazevskaya 42, 127550 Moscow, Russia; a_gulevich@mail.ru; 3 Biology Faculty, Lomonosov Moscow State University, Leninskie Gory 1, Building 12, 119991 Moscow, Russia; 4 Plant Protection Laboratory, N.V. Tsitsin Main Botanical Garden of Russian Academy of Sciences, 127276 Moscow, Russia

**Keywords:** wheat, kernel, caryopsis, maternal tissue, cell layers, terminal differentiation, pericarp, integument, cuticular layer, phenolic layer, nucellus

## Abstract

The integumentary tissues of plant seeds protect the embryo (new sporophyte) forming in them from unfavorable external conditions; therefore, comprehensive knowledge about the structural and functional specificity of seed covers in various plants may be of both theoretical and practical interest. As a result of our study, additional data were obtained on the morphological and ultrastructural features of the formation of a multilayer skin of wheat (*Triticum aestivum* L.) kernel (caryopsis). The ultrastructure research analysis showed that differentiation of the pericarp and inner integument of the ovule leads to the formation of functionally different layers of the skin of mature wheat grain. Thus, the differentiation of exocarp and endocarp cells is accompanied by a significant thickening of the cell walls, which reliably protect the ovule from adverse external conditions. The cells of the two-layer inner integument of the ovule differentiate into cuticular and phenolic layers, which are critical for protecting daughter tissues from various pathogens. The epidermis of the nucellus turns into a layer of mucilage, which apparently helps to maintain the water balance of the seed. Morphological and ultrastructural data showed that the formation of the kernel’s skin occurs in coordination with the development of the embryo and endosperm up to the full maturity of the kernel. This is evidenced by the structure of the cytoplasm and nucleus, characteristic of metabolically active protoplasts of cells, which is observed in most integumentary layers at the late stages of maturation. This activity can also be confirmed by a significant increase in the thickness of the cell walls in the cells of two layers of the exocarp and in cross cells in comparison with the earlier stages. Based on these results, we came to the conclusion that the cells of a majority in the covering tissues of the wheat kernel during its ontogenesis are transformed into specialized layers of the skin by terminal differentiation.

## 1. Introduction

Wheat is one of the most commercially important cereal crops, covering over 30% of world food production. According to Vavilov All-Russian Institute of Plant Genetic Resources (VIR), where almost 100 thousand samples of various cereal crops are stored, seeds of wheat, barley and oat the most durable and capable of maintaining germination capacity. Cereal seeds retain their sowing ability for 5–10 years and keep their biological longevity for more than 30 years [1]. Coat tissues protect seeds from adverse environmental conditions and the effects of extended storage conditions [2,3]. Therefore, a comprehensive knowledge about the structural and functional specificity of the cereal seed coat may be of both theoretical and practical interest.

According to many studies, the caryopsis (kernel) of many cereal plants, including the common wheat *Triticum aestivum* L., has been studied in detail and in many ways. Despite some distinctive features of the structure of kernels of various cereals, the main tissues in them develop without significant differences. A number of studies have presented a detailed analysis of the structure and physiology of specific parts of the kernel, including the pericarp [4,5,6,7], nucellus [8], endosperm [9] and all maternal tissues in general [10,11]. Many authors are of the opinion that coordinated physiological interactions between maternal and daughter tissues are critical for the normal development and maturation of grain [10,12,13,14,15]. It is generally accepted that, in addition to the protective function, the maternal tissues of maturing cereal grains play an important role in the formation and development of daughter tissues, delivery of water and nutrients to the embryo and endosperm; therefore, they affect the growth of endosperm and, accordingly, determine the final seed size [14,15,16]. In addition, seed development requires both growth and differentiation of new tissues, which are coordinated with the controlled disappearance and/or degradation of many maternal tissues through the mechanisms of programmed cell death (PCD) [11,14,16,17,18]. Currently, PCD is considered as a key process that promotes the regulated growth and development of many tissues and organs of angiosperms [19,20]. A number of studies have demonstrated that all maternal tissues of a mature kernel (caryopsis), with the exception of the embryo and the aleurone layer of the endosperm, undergo controlled degradation [13,14,21]. A number of authors presented a detailed light-optical study of the ontogenesis of rice kernel (caryopsis) within 30 days after flowering [11]. They determined that, by the 21st day after flowering, all maternal tissues were already degraded. In addition, similar studies of the kernel development of barley revealed that the PCD of maternal tissues correlates with specific stages of endosperm development [10,22]. Thus, it is currently believed that almost all maternal tissues undergo controlled degradation by PCD. However, it remains unclear how these events manifest themselves at the structure level, which cellular components or derivatives of cells contribute to the formation of the kernel of cereals, how the protective layers are formed and what the specific structural features of each layer are. Thus, the goal of our study was to analyze the successive stages of differentiation of all tissues of wheat kernel using a combination of light and electron microscopy and to determine the functional role and fate of each type of maternal tissue in the formation of integuments that can reliably protect the endosperm and young sporophyte from various harmful environmental influences.

## 2. Results

In this work, the morphological and structural aspects of the process of differentiation of all maternal tissues in wheat (*Triticum aestivum* L.) kernels were studied in detail using light and electron microscopy.

**Figure 1:**
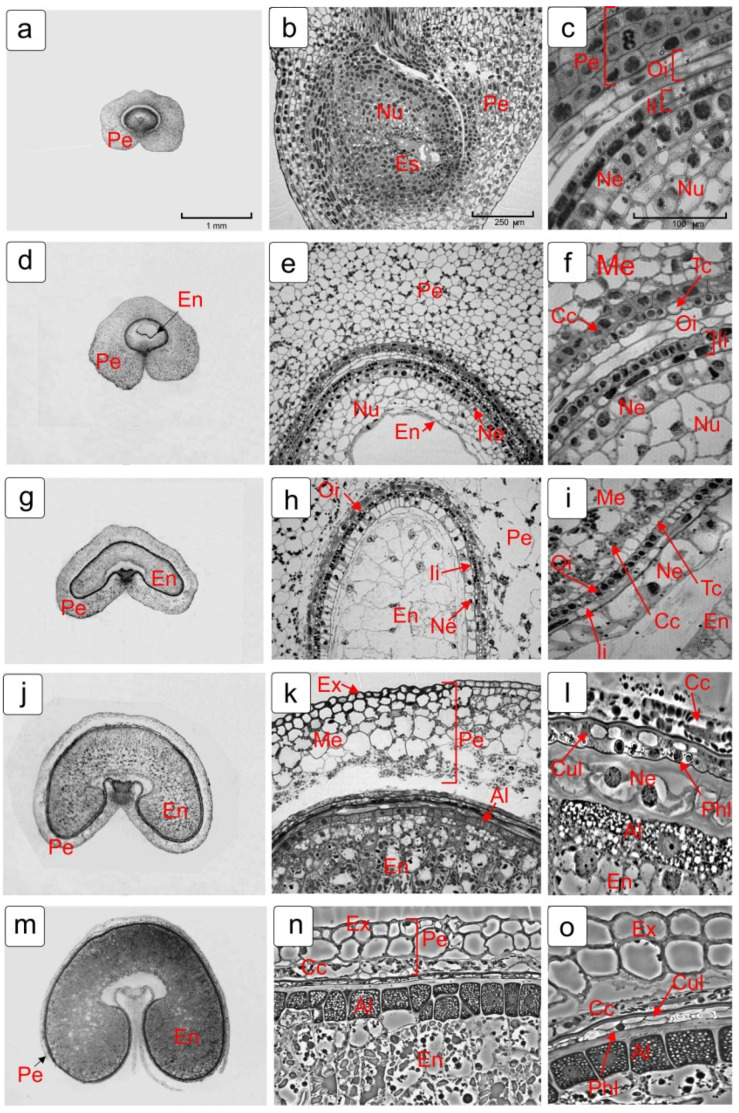
Morphological changes in the tissue structure of wheat kernels during its development and maturation. Different rows show sections of wheat kernel in different stages of development: before fertilization (**a**–**c**); coenocyte stage (**d**–**f**); cellularization stage (**g**–**i**); milk stage (**j**–**l**); soft dough stage (**m**–**o**). Light microscopy of cross sections of wheat kernel—(**a**,**d**,**g**,**j**,**h**,**m**). Longitudinal median section of kernel (**b**) and its fragment (**c**) before fertilization is shown. In other rows, the fragments of transverse sections from the dorsal side of the kernel are seen (**e**,**h**,**k**,**l**,**n**,**o**). Fragments of transverse section from the lateral side of kernel are seen only in (**f**,**i**) images. Abbreviations: Al—aleurone layer; Cc—cross cell; Cul—cuticular layer; En—endosperm; Ex—exocarp; Es–embryo sac; Ii—inner integument; Me—mesocarp; Mc—mucilage cells; Ne—nucellar epidermis; Nu—nucellus; Oi—outer integument; Pe—pericarp; Phl–phenolic layer; Tc—tube cells.

For the study, we used those stages of the ontogenesis of the kernel that coordinate with the key stages of endosperm formation, namely, the stage of the embryo sac before fertilization, the coenocytic stage of the endosperm, the stage of the endosperm cellularization and early stages of its development, as well as the milk and soft dough stages of kernel maturation (Figure 1).

Almost all maternal tissues are involved in the formation of the kernel skin—pericarp, inner integument and epidermis of ovule nucellus.

The changes in the structure of cells in maternal tissues are coordinated with the growth of the kernel. As a kernel develops and matures, the volume of daughter tissues (embryo and endosperm) increases and the volume of maternal tissues, respectively, decreases and narrows markedly (Figure 1). At the same time, some of the maternal tissues are eliminated, while others differentiate further and form specialized integumentary layers.

Figure 1 shows cross sections of wheat kernels in the studied developmental phases (Figure 1a,d,g,j,m). The median longitudinal section of the ovary before fertilization (Figure 1d) and the fragments of cross sections of the kernel in other stages of development upon different magnifications (Figure 1e,h,k,n; Figure 1c,f,i,l,o) are presented. In the early stages of development, the pericarp of wheat kernel contains from 15 to 20 and more layers of cells (Figure 1a,d). After fertilization, because of enlargement of the embryo sac, its thickness decreases to 3–4 layers (Figure 1k) due to the destruction of mesocarp cells and, by the soft dough stage, the mesocarp cells are completely degraded (eliminated) (Figure 1n,o).

The nucellus, in the nuclear endosperm stage, contains 4–5 layers of cells (Figure 1c), but, when the endosperm cellularization occurs, only the outer layer of cells, the epidermis (Figure 1h,i), remains from the nucellus. With further endosperm development, peripheral cells differentiate into the aleurone layer and starch accumulates in the main endosperm (Figure 1k,n).

**Figure 2:**
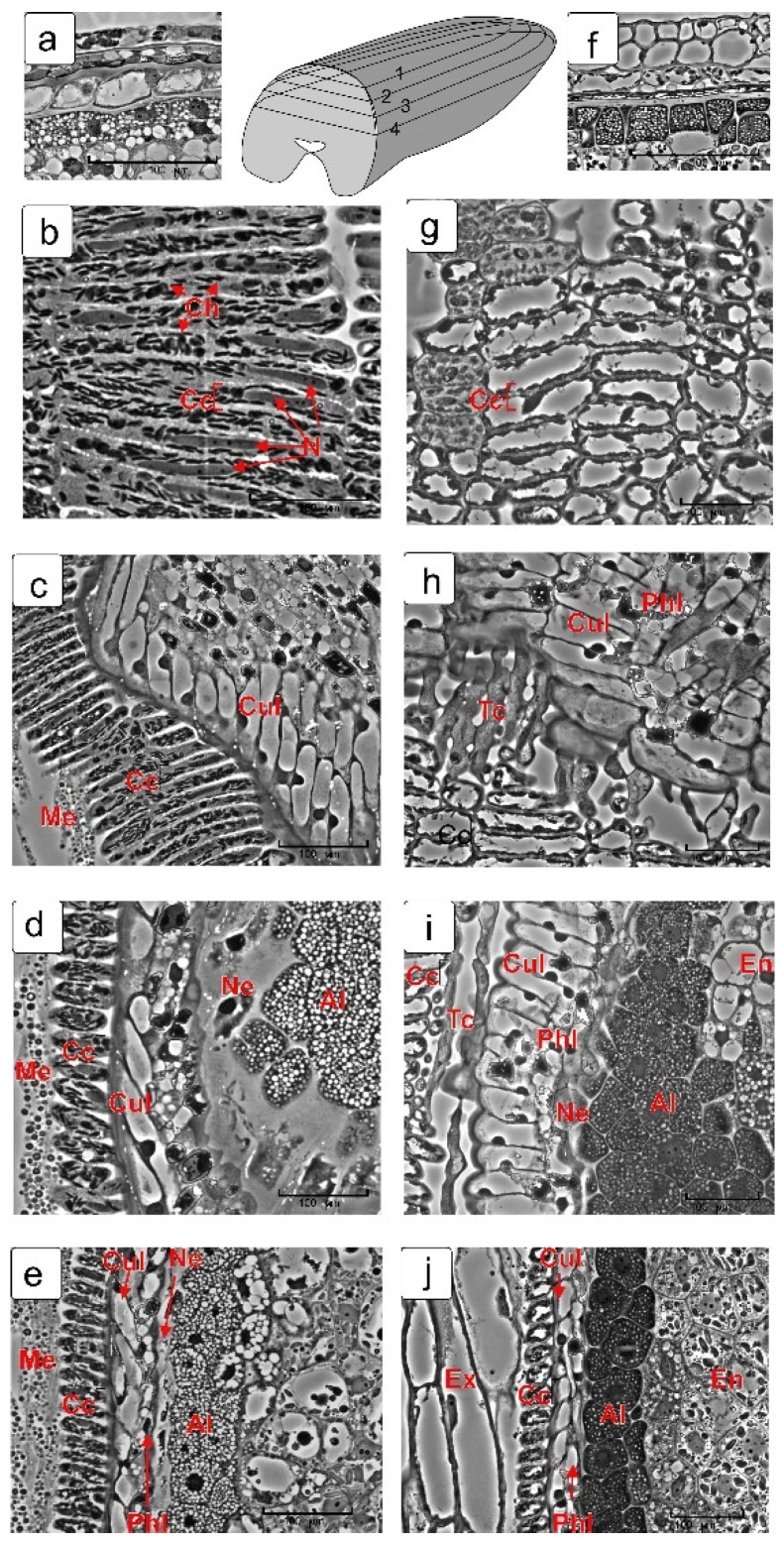
Longitudinal tangential sections from the dorsal side of kernel (schema). Early stage of kernel development (milk stage): transverse section (**a**) and longitudinal section (**b**–**e**). Late stage of kernel development (soft dough stage): transverse section (**f**) and longitudinal section (**g**–**j**). Top row, the middle image—schematic representation of longitudinal serial sections from the dorsal side of kernel. Abbreviations: Al—aleurone layer; Cc—cross cell; Ch—chloroplast; Cul—cuticular layer; En—endosperm; Me—mesocarp; Ne—nucellar epidermis; M—mucous; Phl—phenolic layer; Tc—tube cells.

To better understand the spatial arrangement of maternal tissue cells, longitudinal serial sections were made from the dorsal side of the kernel parallel to its longitudinal axis (Figure 2, top row, scheme).

The cuticular and phenolic layers of the integument in both phases differ somewhat from each other in the mutual arrangement of the cells, but the difference in structure is not noticeable. All cells of the cuticular layer contain the nuclei that may indicate that they are alive. The cells of the phenolic layer also contain nuclei, but they are not always visible due to the large number of phenolic clots of different sizes (Figure 2c,h).

The epidermal cells of the nucellus are not always clearly visible due to mucilage in them. Only in the milk stage on the tangential cut, the cells themselves and the nuclei in them are visible (Figure 2d) and, in the late phase in the site of the nucellar epidermis, only a strip of mucilage is visible (Figure 2i), since, by this time, the protoplasts of these cells have degraded. Sections made in the middle part of the kernel (Figure 2e,j) show the longitudinal sections of cells in all layers of maternal tissues and during the two stages of endosperm formation.

In order to better understand the structural and functional features of maternal tissues that form the skin of wheat kernels, a study of the cell ultrastructure of all these tissues during their formation and development was carried out.

### 2.1. Pericarp

Wheat pericarp develops from the ovary wall and is successively divided into exocarp, mesocarp and endocarp [4]. Tube cells constitute the actual endocarp (the inner epidermis of the pericarp) [11]. However, most researchers studying the sub-epidermal chlorophyll-bearing layer (cross cells) also refer to the endocarp.

**Figure 3:**
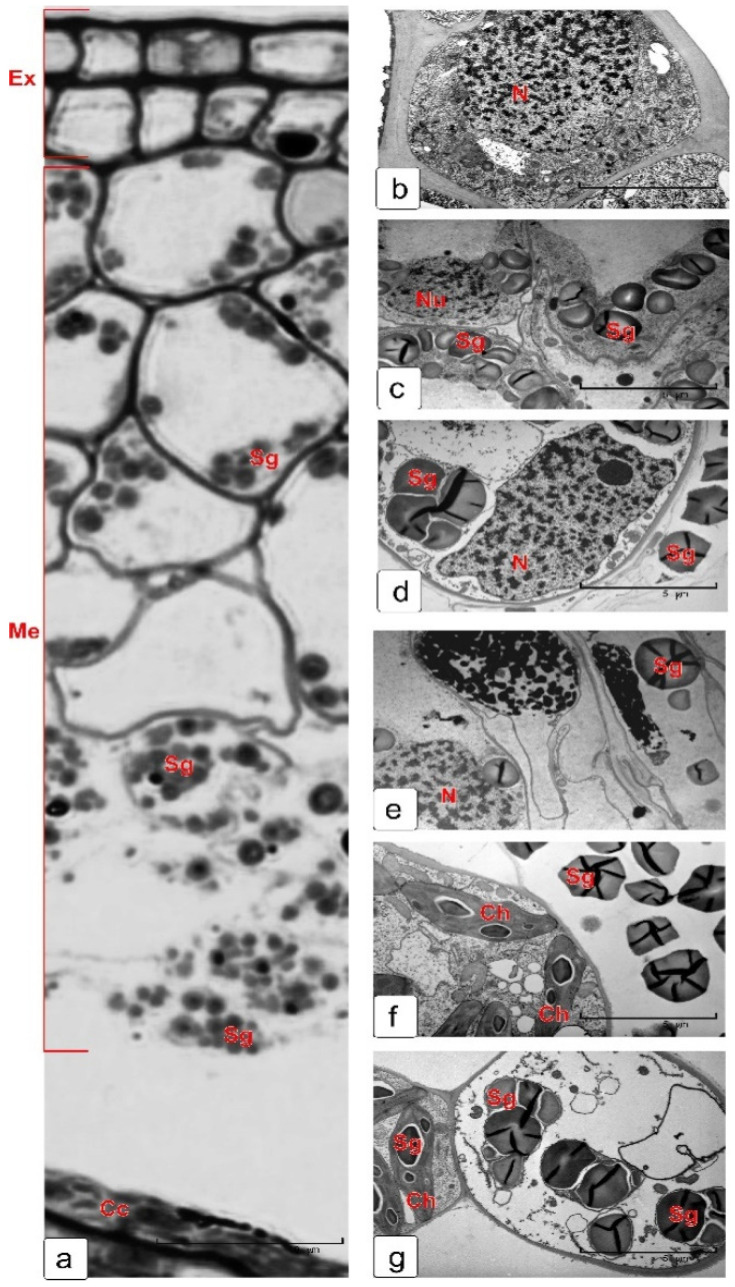
Cross section fragment of wheat kernel pericarp in the early milk stage (**a**) and ultrastructural fragments of cells from different zones: exocarp cell (**b**), cells near the exocarp (**c**,**d**), disintegrating mesocarp cells in the zone of cross cells (**e**), released starch grains near by the cross cell (**f**), a degrading cell still connected to the cross cell (**g**). Legend: Cc—cross cell; Ch—chloroplast; Ex—exocarp; Me—mesocarp; Sg—starch grain.

Figure 3 shows the fragments of exocarp and mesocarp cells in a cross section of a kernel in the middle stage of endosperm formation, close to milk stage (Figure 3a).

Exocarp cells (Figure 3b) have a rectangular or oval shape and thickened walls that are characteristic of integument tissues, which perform a protective function. They contain a large nucleus and a dense cytoplasm saturated with numerous organelles.

In this stage of development, dynamic changes occur in the mesocarp. They are associated with the process of starch hydrolysis and cell destruction. This happens in different ways in different layers of the mesocarp. The cells located near the exocarp (Figure 3c) looked quite intact; they contain a large amount of starch grains in the amyloplasts that have begun to break down. In the central layers of the mesocarp, cells with varying degrees of PCD can be also observed in them (Figure 3d,e). Next to the chlorophyll-bearing layer (cross cells), only starch grains of different size "dropped" from the cells can be observed (Figure 3f). An almost completely destroyed (degraded) cytoplasm and half-destroyed plastids with starch grains inside (Figure 3g) can be observed in the mesocarp cell, still connected to the cross cell.

### 2.2. Cross Cells (Chlorophyll-Bearing Layer)

**Figure 4:**
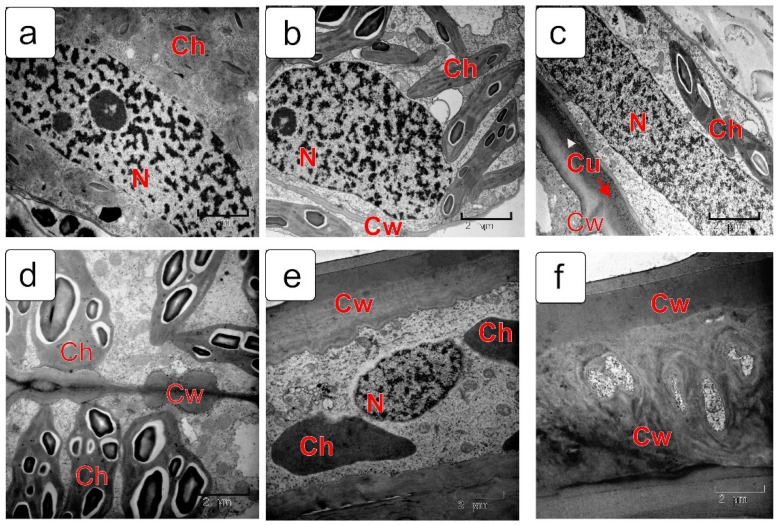
Ultrastructure of cross cells in different stages of kernel development: coenocytic (nuclear) endosperm stage (**a**); endosperm cellularization stage (**b**); early milk stage of endosperm filling (**c**,**d**); soft dough stage (**e**,**f**). Abbreviations: Ch—chloroplast; Cw—cell wall; N—nucleus.

Some stages of differentiation of chlorophyll-bearing (cross) cells are presented in Figure 4. In the nuclear endosperm stage (Figure 4a), a very dense cytoplasm, large nuclei and numerous juvenile chloroplasts with small grana and small starch grains are observed in cross cells. By the time the cell endosperm is formed, chloroplasts noticeably increase in size and their structure becomes more complex (Figure 4b). As the grain grows further, the cross cells are extended in length and the nucleus in them also acquires an elongated shape (Figure 4c). By the milk stage, the envelopes of the cross cells thicken unevenly (Figure 4d) and, by the soft dough stage of the kernel, the thickness of the envelopes increases even more and takes the shape of a spiral (Figure 4e,f). By this time, the structure of the cytoplasm and chloroplasts is noticeably simplified and the starch disappears.

### 2.3. Endocarp (Tube Cells)

**Figure 5:**
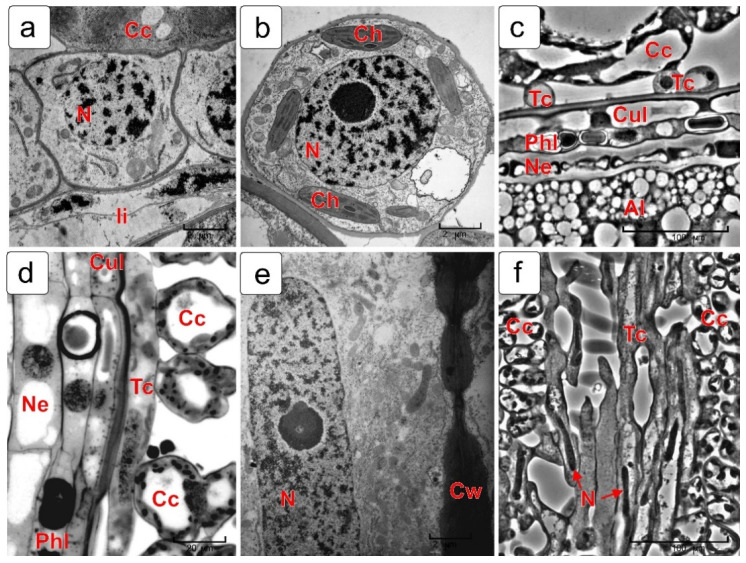
Formation of tube cells in wheat kernel. Transverse (**a**–**c**) and longitudinal (**d**–**f**) sections from a light and electron microscopy are presented. Coenocytic endosperm stage (**a**); endosperm cellularization phase (**b**,**d**); milk and soft dough stage (**c**,**e**,**f**). Legend: c—cross cell; Ch—chloroplast; Cul—cuticular layer; Cw—cell wall; N—nucleus, Ne—nucellar epidermis; Phl—phenolic layer; Tc—tube cells.

Figure 5 shows the differentiation of endocarpic cells or tube cells. In the stage of nuclear endosperm stage, the cells are quite tightly adjacent to each other and to the degrading cells of the outer integument (Figure 5a). During the formation of cellular endosperm, the endocarp cells are strongly elongated in the longitudinal direction (Figure 5d) and, in the cross section, they acquire a rounded shape (Figure 5b). In this stage, most of the tube cells detach from each other and, as the kernel increases in volume, they move away from each other (Figure 5c). In the milk and soft dough stages, tube cells contain an elongated nucleus and a rather dense cytoplasm, while their membranes are unevenly thickened (Figure 5e,f).

### 2.4. Integuments of Ovules

#### 2.4.1. Outer Integument of Ovule

**Figure 6:**
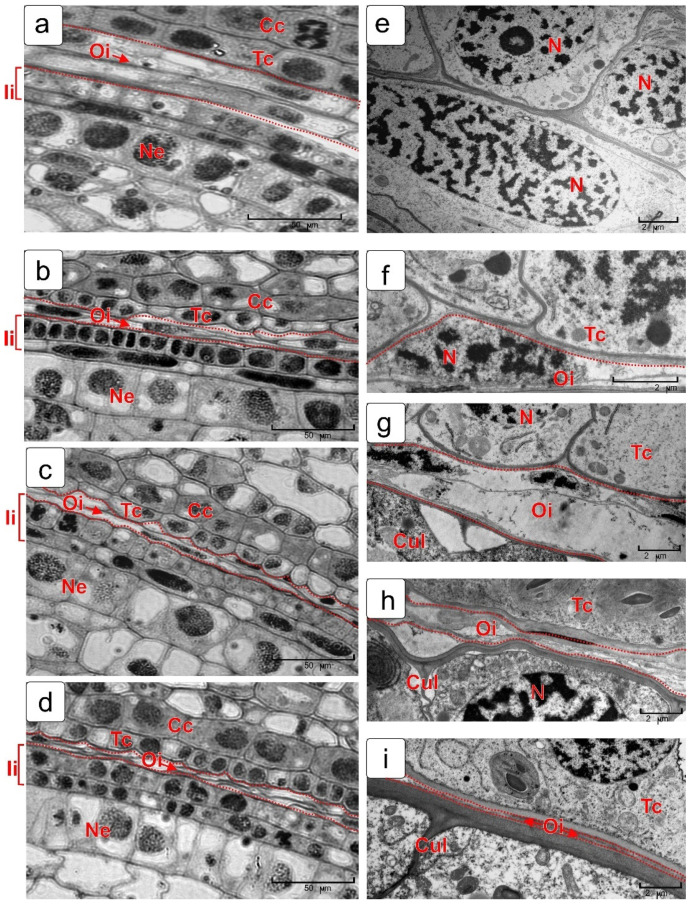
Process of the outer integument death in wheat kernel. Images from light (**a**–**d**) and electron microscopy (**e**–**i**) are presented. Legend: Cc—cross cell; Cul—cuticular layer; Ii—inner integument; Oi—outer integument; Ne—nucellar epidermis; Tc—tube cells.

The outer integument of the ovule in the wheat kernel disappears completely by the period of cell formation in the endosperm. The PCD process in outer integument cells is shown in Figure 6 in semi-thin (Figure 6a–d) and ultrathin (Figure 6e–i) sections. Even before fertilization, the cells of the outer integument differ from the cells of the inner integument by a lighter cytoplasm (Figure 6a). Immediately after fertilization, the cells stop dividing and only nuclei are visible in these cells in sections against a light background. In ultrathin sections, it can be seen that, first, due to lysis, the cytoplasm of these cells (Figure 6f,g) disappears; then, the nucleus disappears without noticeable structural changes inside of these cells. Only the envelopes (Figure 6h) remain from the cells of the outer integument, as well as a narrow strip of them between the cuticular layer of the integument and tube cells (Figure 6i).

#### 2.4.2. Inner Integument of Ovule

**Figure 7:**
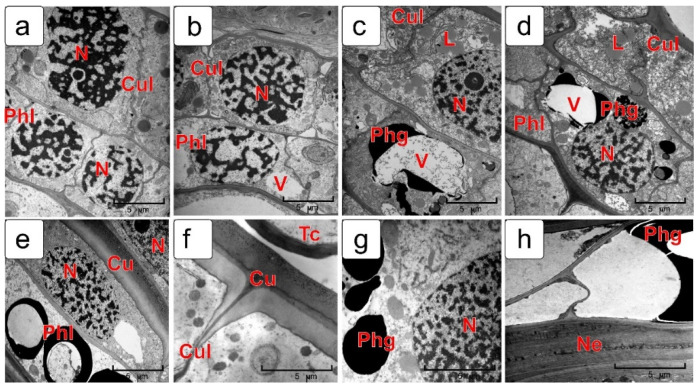
Differentiation process of two layers of the wheat ovule inner integument. Pre-fertilization stage (**a**); coenocytic endosperm stage (**b**); endosperm cellularization stage (**c**,**d**); milk stage (**e**,**f**); soft dough stage (**g**,**h**). Abbreviations: Cu—cuticle; L—lipid droplets; N—nucleus; Ne—nucellar epidermis; Phg—phenolic grain; Tc—tube cells; V—vacuole.

The inner integument of the ovule also consists of two cell layers (Figure 7). At the early stages of development, the cells of both layers have a rectangular shape and differ little in their internal structure, although the cells of the outer layer are usually larger (Figure 7a,b).

Further differentiation of each layer occurs in completely different ways (Figure 7c,d). This is clearly seen in the early cellular endosperm stage. The metabolism of the outer (cuticular) layer is directed to a formation of a thick cuticle on the outer cell walls bordering the exocarp. A characteristic feature of these cells is the presence of a large number of lipid droplets in the cytoplasm. The inner layer of the inner integument is differentiated by the pathway of synthesis and accumulation of phenolic compounds, which are deposited both in the vacuoles and in the cytoplasm. They are clearly visible in cell sections in form of coarse—or fine—granular clusters or bodies of various sizes (Figure 7c–e,g).

When the endosperm in the kernel reaches the milk stage, the outer coats of the cuticular layer are covered with a thick cuticle (Figure 7f) and a large number of phenolic compounds (Figure 7e,g,h) accumulates in phenolic layer cells. Moreover, in most cells of both layers of the integument, undegraded cytoplasm is observed (Figure 7f,g).

### 2.5. Epidermis of Nucellus

**Figure 8:**
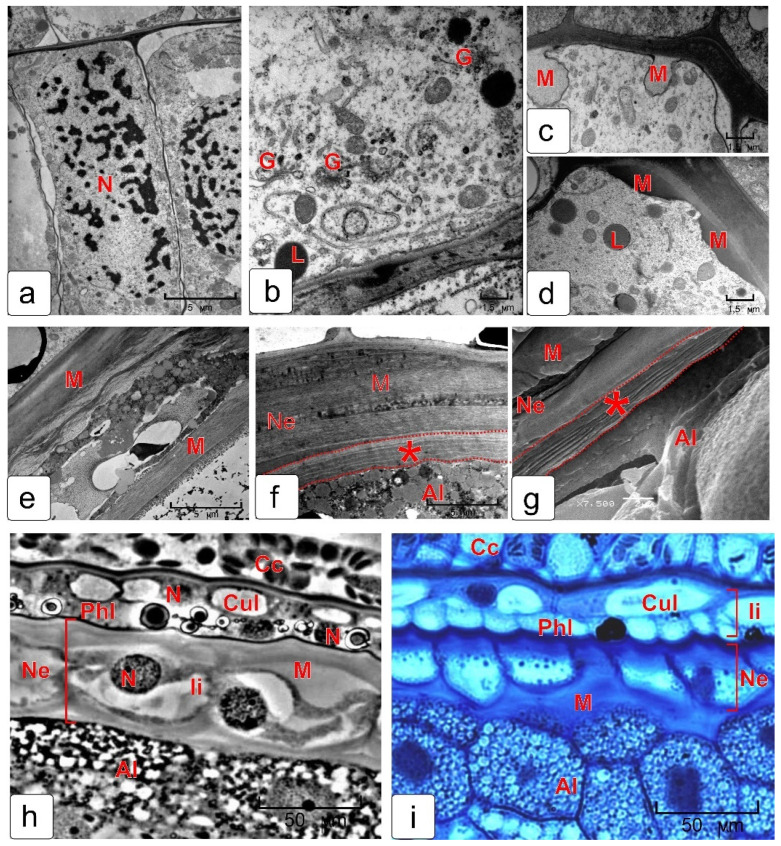
Differentiation of the epidermis of nucellus cells and their death by mucilage (deposits of pectin mucilage) (**a**–**g**). Nucellus epidermal cells in the nuclear endosperm stage (**a**). A fragment of a cell of the epidermis of the nucellus with a highly developed Golgi apparatus, which is characteristic of mucilage-forming cells (**b**). Mucilage is gradually deposited between the plasmalemma and the cell membrane (**c**,**d**); the protoplast is compressed (**e**) and dies (**f**). The same fragment was made on a scanning microscope (**g**). The structure of the cells of the epidermis of the nucellus on semi-thin (light-optical image) sections in the process of their mucification (**h**,**i**). Pectins were identified by staining the preparation with toluidine blue (I). Abbreviations: Al—aleurone layer; Cc—cross cells; Cul—cuticular layer; G—Golgi; L—lipid droplets; M—mucilage; N—nucleus; Phl—phenolic layer; The asterisk denotes the layers formed from the remnants of the membranes of the integument parenchyma cells that died by PCD. These layers strengthen the outer cell walls of the aleurone layer of the endosperm.

The nucellus is the body of the ovule, where an embryo sac is formed and then a new sporophyte is developed. Daughter tissues (embryo and endosperm) formed after fertilization begin to grow and nucellus cells die due to PCD. Some stages of the mucilage (or jelly-like) layer formation of the kernel skin by nucellar cells are presented in Figure 8. In the phase of nuclear endosperm, the nucellus consists of 3–4 layers of cells and epidermis. In the cross section of the kernel, the cells of nucellar epidermis and their large nuclei are elongated perpendicular to the integument (Figure 8a). As the ovule grows, the shape of cells changes to round. Feature of the cytoplasm of nucellar epidermis cells is a highly developed Golgi apparatus (Figure 8b), which is characteristic of mucilage-secreted cells. Mucilage is unevenly deposited in the space between plasma membrane and cell wall of the nucellus epidermis (Figure 8c,d,h,i). The pectin composition of mucilage was determined by staining semi-thin sections with metachromatic dye toluidine blue [23]. The mucilage-produced cells remained alive almost until the milk stage of kernel filling (Figure 8h,i). However, the cytoplasm gradually died (Figure 8e) and the cells were completely filled with mucilage (Figure 8f). In the inner side, this layer is in contact with the thickened outer cover of the aleurone layer of endosperm, which was formed as a result of the fusion of the outer envelopes of endosperm cells and non-lysed walls of dead nucellus cells (Figure 8f). These layers are clearly visible in the image obtained with scanning microscopy (Figure 8g).

### 2.6. Endosperm

**Figure 9:**
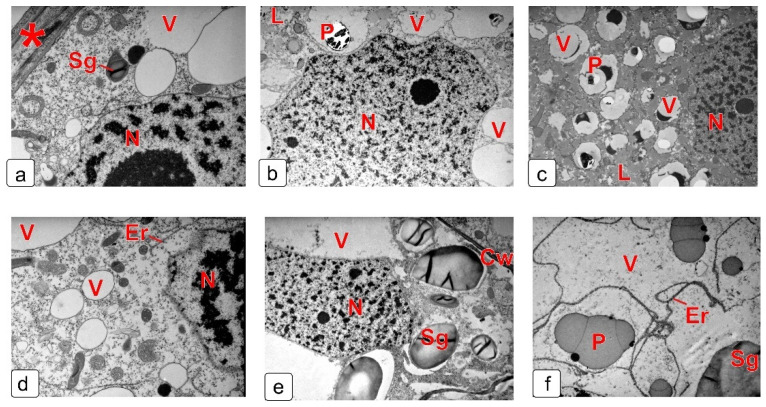
Fragments of cells of the aleurone layer (**a**–**c**) and the main (starchy) endosperm (**d**–**f**) at some early stages of wheat kernel formation. (**a**,**d**) cellular endosperm stage; (**b**) beginning of differentiation of the aleurone layer; (**c**,**e**,**f**) milk stage; (**e**) main endosperm cell next to the aleurone layer; (**f**) main endosperm cell in the deeper layers. Abbreviations: L—lipid droplets; N—nucleus; P—protein body; Sg—starch grain; V—vacuole; * envelope remnants of nucellus cells.

The expansion and differentiation of the endosperm in the kernel of wheat is activated after the transition from the nuclear phase to the cellular phase. During this period, the endosperm borders on the nucellus’ epidermis, but some space remains between them, filled with remnants of the cell envelopes of the dead nucellar parenchyma (Figure 9a). At this stage, the structure of cells in the peripheral zone and more internal layers is practically the same (Figure 9d). This period is characterized by the presence of small, rounded vacuoles in the cytoplasm (Figure 9a,d). At the milk stage, the forming protein bodies inside the vacuoles and a large amount of lipid drops in the cytoplasm accumulate in the aleurone cells (Figure 9c). In the main endosperm, an increase in the size of starch grains was observed, as well as the formation of protein bodies (Figure 9e,f).

**Figure 10:**
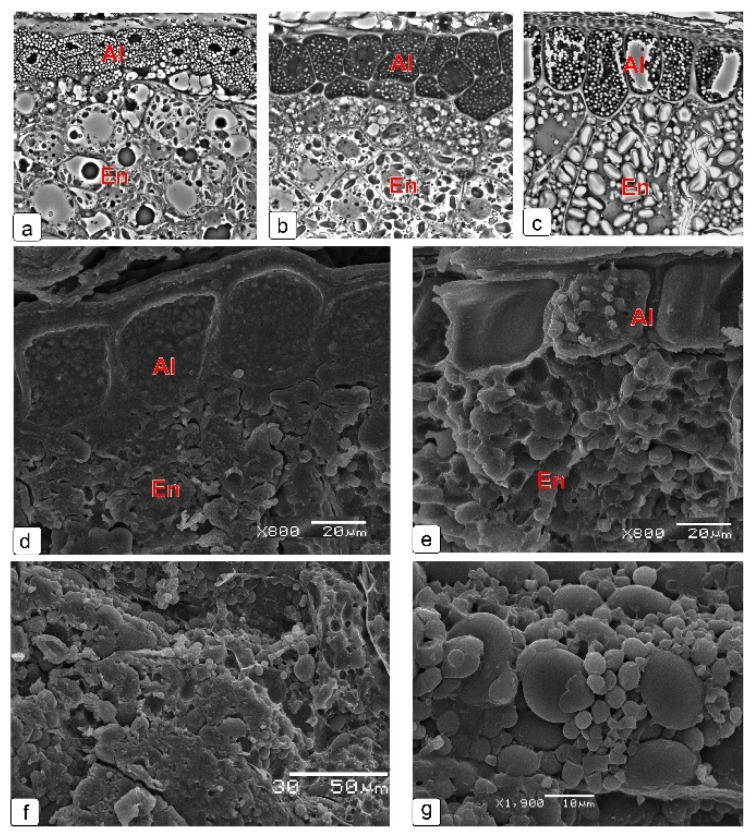
Structure of wheat kernel endosperm at the late stages of kernel maturation on a light-optical microscopy (**a**–**c**) and on a scanning electron microscopy (**d**–**g**); (**a**) milk stage; (**b**,**d**,**f**) soft dough stage; (**c**,**e**,**g**) stage of full maturity (dry kernel). Abbreviations: Al—aleurone layer; En—endosperm.

At the later stages of endosperm development (Figure 10), there is a gradual thickening of protein globules in the aleurone layer. In addition, a decrease in the size and number of protein bodies is observed in the starchy (main) endosperm (Figure 10a–c). At the final stage of kernel development, starch is a heterogeneous fraction, which is clearly seen on the scan image (Figure 10g).

### 2.7. Cell Structure of All Layers of Kernel Skin at Final Stage of Development

**Figure 11:**
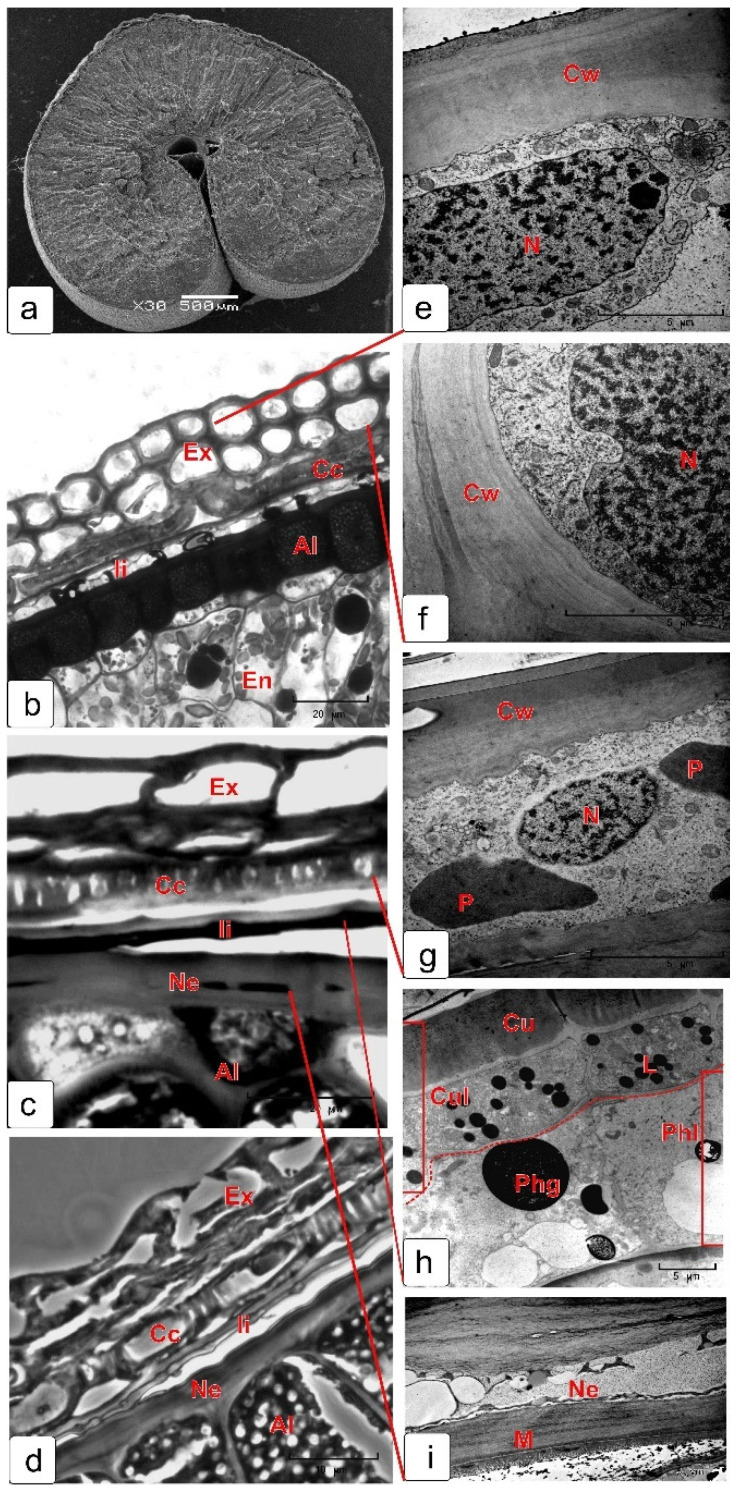
Skin structure of mature and dehydrated wheat kernel. General cross-sectional view (**a**); skin layers before dehydration (**b**); dry, but slightly soaked layers before cutting (**c**,**d**). Ultrastructure of cells forming the kernel’s skin in final maturation stage (before dehydration): cell of exocarp outer layer (**e**), cell of exocarp inner layer (**f**), cross cell (**g**), cells of integument cuticular and phenolic layers (**h**), mucilage cell layer (**i**). Abbreviations: Al—aleurone layer; Cc—cross cell; Ch—chloroplast; Cu—cuticle; Cul—cuticular layer; Cw—cell wall; En—endosperm; Ex—exocarp; Ii—inner integument; L—lipid droplets; Mc—mucilage cell; Ne—nucellar epidermis; N—nucleus.

Figure 11 shows the structure of the skin of a mature kernel (Figure 11a), all its layers before dehydration (Figure 11b) and after dehydration (Figure 11c,d), as well as the ultrastructure of the cells of each layer before dehydration (e–i). Degraded cytoplasm can be observed only in the cells of nucellar epidermis, which form the mucilage layer.

**Figure 12:**
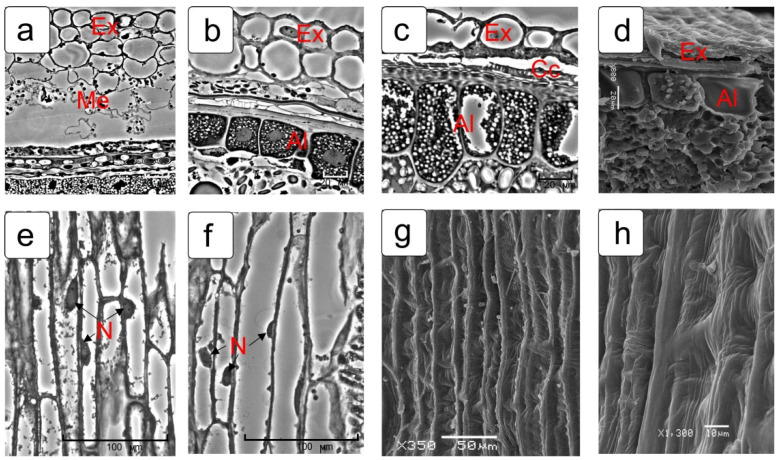
The process of the kernel’s outer skin formation by exocarp cells. Cross sections (**a**–**d**), longitudinal sections (**e**,**f**) and surface view (**g**,**h**). Stages: milk (**a**,**e**), soft dough (**b**,**f**), full maturation (**c**,**g**,**d**,**h**). Scanning electron microscopy images are presented (**g**,**d**,**h**). Abbreviations: Al—aleurone layer; Cc—cross cell; Cul—cuticular layer; Ex—exocarp; Me—mesocarp; En—endosperm; Ne—nucellar epidermis; Phl—phenolic layer; Ii—inner integument.

Figure 12 shows the process of formation of the outer texture of the kernel’s skin by the exocarp cells. Fragments of transverse (a–d) and corresponding longitudinal sections (Figure 12e,f) of the surface layers of the kernel at different stages of maturation, as well as a view of the skin of a mature kernel from the surface (Figure 12g,h), obtained by scanning microscopy, are presented.

The confirmation of the long-term maintenance of the viability of exocarp cells (the outer layer of the pericarp) was given by the preservation of the following: (1) fine structure of the cytoplasm, until the grain is completely filled; (2) preservation of the structural organization and integrity of the organelles and their surrounding membranes; (3) preservation of the structural organization of the nucleoplasm and the structures of hetero- and euchromatin, as well as the cell wall characteristics of actively metabolizing cells. The figures show changes in the exocarp cells from the initial stage to the complete filling of the kernel, accompanied by a thickening of the cell wall (Supplementary Materials, Figure S1).

The cross cells of the pericarp at the milk stage of kernel maturation were well identified as actively photosynthetic cells. They contained a large oblong nucleus located in the center of cell, surrounded by numerous plastids–chloroplasts (Supplementary Materials, Figure S2a). The fine structure of these cells revealed highly dispersed chromatin, characteristic nucleoli, plastids containing stacks of grana, dense stroma and starch grains, mitochondria having a dense matrix and pronounced cristae (Supplementary Materials, Figure S2b). At the soft dough stage, the cross cells of pericarp contained clearly identified large nuclei; the plastids were located mainly along the cell wall, which was caused by a high degree of vacuolization (Supplementary Materials Figure S2c). These cells also contained an active cytoplasm and many mitochondria with pronounced cristae, while the nuclei also retained an active nucleoplasm with pronounced euchromatin fragments and heterochromatin regions. Plastids changed their structural organization, the grana were not detected and their structure corresponded to leukoplasts (Supplementary Materials, Figure S2d). A significant increase in the thickness of the cell walls occurred by the completion of the grain filling. Subsequently, this manifested in the formation of a very solid texture due to complication of the deposition of additional layers of the cell wall (Figure 4d–f). Thus, these cells retained their viability at the late stages of kernel formation until the grain was completely filled and until the beginning of dehydration. In the process of dehydration of a mature kernel, the external texture of its skin is formed. Two layers of the exocarp are known to be involved in this process. These two layers consist of large, longitudinally elongated cells, as well as cross cells. According to anatomical data, the cross cells, before kernel dehydration, bended in the form of arcs and attached to the exocarp cells by their ends (Figure 10b). The middle parts of the cross cells were drawn inward and the ends attached to the exocarp cells formed a longitudinal ribbing of the kernel skin under dehydration (Figure 11b). This longitudinal ribbing did not look very level due to the varying length of both the cross cells and the cells of the exocarp itself. Tube cells were present sparsely, unevenly and not in all the parts in a mature kernel and, apparently, did not participate in the formation of the texture of the skin surface. Some authors have noted that tube cells with thick walls can provide rigid support in kernels [13]. We assume that the differentiation of these pericarp tissues also occurs by the type of terminal differentiation.

## 3. Discussion

In this work, we analyze, in detail, the structural changes in the cells of the maternal tissues of wheat kernel during its maturation. The study shows that all maternal tissues of wheat kernel (caryopsis) follow distinct morphological pathways of differentiation, which leads to the formation of its multifunctional coat (skin). Based on the results obtained, we came to the conclusion that the main direction of differentiation of most maternal tissues of wheat kernel is the formation of functionally different layers to protect the developing embryo (new sporophyte) and its long-term preservation in mature grain. Previous studies have shown that in the ontogenesis of cereal grains, as in the development of seeds of other plants, some tissues are programmed for complete death, but this happens in different ways, and this death has different causes and meanings. Such tissues in the kernel include cells of the outer integument of the ovule, parenchymatous cells of nucellus and cells of mesocarp. Of these, first and almost simultaneously (shortly after fertilization), the cells of the external integument and parenchyma of nucellus are degraded. The death of the mesocarp cells of the pericarp also begins after fertilization, but it stretches in time almost to the soft dough stage (wax ripeness) of the kernel, as has been shown in other cereals [10,11]. Here, we studied mainly the tissues that form the main layers of the kernel skin in detail.

### 3.1. Pericarp

**Figure 13:**
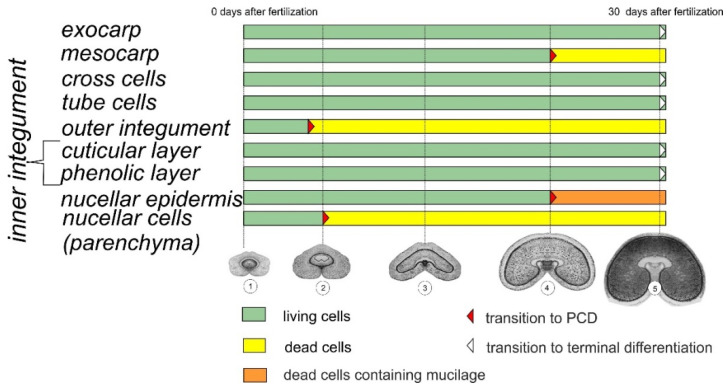
Schematic drawing of maternal tissue differentiation during formation of the coat in wheat kernel. Green box—living cells with fully functional cellular organelles; yellow box—dead cells; orange box—dead cells filled with the mucilage; red arrowhead—transition to PCD; white arrowhead—transition to terminal differentiation. The time bar indicates the approximate time (days) from the onset of fertilization until the full maturation of the kernel. The key stages of endosperm development (1–5) were used as reference points for identification of the stages of differentiation in other tissues (listed on the left). Stages of endosperm development: (1) prior to fertilization, (2) the stage of syncytial endosperm, (3) the stage of cellular endosperm, (4) the milk stage and (5) the stage of soft dough.

The functional role of structural and physiological changes in the pericarp has been analyzed in detail by many researchers in wheat [4,5,7], barley [10,24,25], rice [11,26], sorghum [6,27] and maize [28,29]. The process of formation, functioning and degradation of mesocarp cells has been studied in particular detail [7,10]. It was shown that the mesocarp plays an important role in the formation of the kernel and, first of all, in the supplying of the endosperm and the developing embryo [4,5]. In our study, the morphological and ultrastructural features of differentiation of other layers of the pericarp—exocarp, chlorophyll-bearing layer (cross cells) and endocarp (tube cells)—were analyzed in detail (Figure 13). The results obtained show that the differentiation of these tissues occurs mainly along the pathway of thickening of the cell walls. Such a modification usually happens due to the deposition of lignin in them. This polymer strengthens the cell wall and reduces its permeability [14,16]. The ultrastructure analysis showed that viable protoplasts and nuclei in the cells of these layers of the pericarp were present until the full maturation of kernel. Convincing confirmation of this viability was a noticeable gradual increase in the thickness of the cell walls in these cells during development and until the kernel fully matures (Figure 11).

### 3.2. Outer Integument

The endocarp of the pericarp is rather tightly adjacent to the outer integument of the ovule in the juvenile stages of kernel development (before fertilization). The outer integument of the ovule in cereals it is known to disappear completely by the time of endosperm cellularization [10,15]. Our study shows that the destruction of the cells of outer integument begins even before fertilization. The ultrastructure analysis revealed that the cytoplasm and cell nuclei of both integument layers disappear as a result of the lytic process after fertilization. This type of cellular destruction and elimination may be induced by mechanical stress [14,16]. It is possible that the fast-growing cells of the endocarp and inner integument exert pressure on the cells of the outer integument, which leads to the destruction of the cytoskeleton in these cells and, ultimately, triggers their death. As a result, after some time, only a thin layer of flattened cell walls remains between the pericarp and the ovule. This compressed amorphous layer probably contributes to the free multidirectional differentiation of tube and cross cells. It is at this time the tube cells begin to elongate along the longitudinal axis of the kernel, acquire a tubular shape and move away from each other, while the cross cells and the cells of the cuticular layer of inner integument elongate in the transverse direction. As the kernel develops, the tube cells move further apart from each other, forming a scaffold-like structure between the pericarp and the ovule [13]. Due to this, they subsequently develop in a coordinated manner, but independently of each other.

### 3.3. Inner Integument

Not so much is known about the structure, differentiation and functional significance of the inner integument cells of the ovule in cereal kernel. It has been reported that cells of the inner integument undergo PCD, thereby providing space and nutrients for the growth of daughter tissues [10,11]. Therefore, we paid special attention to this issue. The structure of cells of both layers of the internal integument at different stages of their differentiation was studied in detail. Our research study shows that these two layers are closely connected to each other. Moreover, although they differentiate simultaneously, they do so in completely different ways. During differentiation, the cells of outer layer of inner integument deposit thick cuticle above the cellulose cell walls. These cells form the so-called cuticular layer of integument. It was previously shown that the cuticle forms a physical barrier and protects cells against drying out and other adverse conditions [30]. According to our ultrastructural data obtained, an increase in the size of the cuticle in these cells occurred almost until the full maturation of the kernel, as evidenced by the undegraded protoplasts and nuclei in these cells at the final stages of development. The differentiation of the inner layer of the inner integument is associated with the synthesis and deposition of phenolic compounds, which gradually accumulate in the cytoplasm of these cells. Phenolic compounds are known to be necessary for plant resistance to pathogens and are involved in many other functions [16,31]. The ultrastructure analysis clearly demonstrated that protoplasts in both layers of the inner integument of ovule retained the morphological features of living cells until the soft dough stage (waxy ripeness) of the kernel. Thus, they play an important role in the development of the kernel, protecting the growing new sporophyte from various pathogens and adverse environmental conditions. Based on the results obtained, we came to the conclusion that this kind of differentiation probably occurs not along the pathway of the PCD, but along the pathway of the so-called terminal differentiation, which is a specialized type of differentiation. During this process, there is the formation of cells of a specific morphology that perform certain functions without undergoing elimination.

Terminal differentiation is an irreversible transition/exit of cells from the cell cycle, consistent with tissue-specific expression of genes characteristic of highly specialized cell types. Finally differentiated cells cannot re-enter the cell cycle, acquire certain features and functions and undergo successive stages of cellular aging and death in the body [32]. Terminal differentiation is very important for the development, maintenance and functional specialization of cells in all multicellular organisms (for example, many types of animal cells). However, the definition of terminal differentiation is rarely used to characterize the life cycle of plant cells; therefore, this issue suggests further research [33].

### 3.4. Nucellus

The most important part of any ovule is the nucellus, especially at an early stage of development, since the mother cell of the megaspore is formed in the nucellus and the first stages of a new sporophyte development take place. The nucellus of the ovule in wheat kernel, as in other cereals, begins to degenerate immediately after fertilization, thereby providing space for growth of the embryo and endosperm [16,18,20,34]. During the development of the kernel and the growth of the endosperm, there is a gradual degradation of the cells of the nucellus parenchyma (from the inner layer to the outer layer). By the time of the endosperm cellularization, only the epidermis remains from the nucellus, as well as the flattened remnants of dead cells of the nucellar parenchyma. These remnants form a kind of buffer layer between the mucous epidermis of nucellus and the endosperm (Figure 8b). The epidermis of the nucellus is then transformed into specialized tissue.

### 3.5. Epidermis of Nucellus

There is no consensus among researchers on the structural and functional significance of this tissue. According to some researchers, the epidermis of the nucellus consists of cells with thick cell walls and enriched with lipids [11,13]. It is assumed that these highly specialized cells can provide a water-resistant barrier that isolates the endosperm from the integuments. However, the results of our ultrastructure study showed that there was no apparent thickening of the cell walls in cells of the nucellus epidermis. In contrast, there was a gradual accumulation of a mucus-like substance containing pectins between the primary cell wall and the plasma membrane. Pectocellulosic mucilage, which is usually synthesized by the cells, is deposited between the primary cell wall and the plasma membrane. This leads to the pushing back of the protoplast from the cell wall to the center of cell. The protoplast gradually degrades and disappears by the stage of soft dough (waxy ripeness). A similar process of mucilage formation has been described for integument cells in the caryopsis of *Hieracium*, *Pilosella* and other members of the *Asteraceae* family [23,35,36]. In our study, the pectin composition of the mucilage was confirmed by staining of semi-thin sections with toluidine blue [23]. In the kernel of wheat and other cereals, mucilage-producing cells of the nucellus epidermis are likely to play an important role in protecting the embryo and endosperm from drying out, since mucilage is able to retain large amounts of water. It should also be noted that the replacement of the protoplast with mucilage, observed in the epidermis of nucellus and in other similar plant cells, can be considered as one of the variants of ontogenetic PCD [20]. The mucilage-filled cells of the nucellus epidermis are the innermost layer of the kernel skin, in direct contact with the embryo sac. As a rule, there is a layer of flattened envelopes of degraded cells of the nucellar parenchyma between the endosperm and the epidermis of nucellus. These layers of residual envelopes are clearly visible in the images obtained from transmission and scanning electron microscopy even at late stages (Figure 8f,g).

### 3.6. Endosperm

The expansion and differentiation of the endosperm in the kernel of wheat is activated after the transition from the nuclear stage to the cellular stage. At a later stage, the aleurone layer and the main (starchy) endosperm differentiate. It is generally accepted that, in a mature cereal kernel, all cells of the starchy endosperm die by PCD and only the cells of the aleurone layer remain alive [9,17,37]. There is also a study showing that PCD in wheat endosperm is just a random process [38]. In rice endosperm, PCD occurs simultaneously with the rapid accumulation of starch grains, which, according to researchers, indicates that PCD does not affect starch biosynthesis [15]. The authors suggested that, most likely, PCD in rice endosperm causes only partial degeneration of cytoplasmic membranes, while the structure of the nucleus and organelles remain functional during this process of cereal endosperm development, allowing starch biosynthesis to occur in a large endosperm compartment with a common cytoplasm. However, the data of light (especially tangential sections of the kernel (Figure 2)) and electron microscopy showed that the cells of both the aleurone layer and the starchy endosperm retained all the structural elements of metabolically active cells until the full maturity of the wheat kernel. With the exception of the final stage—the stage of dehydration—this is necessary for long-term preservation.

## 4. Materials and Methods

### 4.1. Plant Material

Winter wheat *Triticum aestivum* L. (cv Moskovskaya 39; 2n = 42) was used in a field experiment on the territory of the RSAU Moscow Agricultural Academy (Moscow, Russia) from 10 June to 25 July in 2019 and 2020. The weather conditions were typical for the given climatic zone. The selection began one day after flowering and continued until full maturation. Ears were taken for research analyses at an interval of 5–7 days. The differences in plant development were negligible. The ears with part of the stem were cut off, placed in a vessel with water and delivered to the laboratory. Ovules with the desired development stages were selected under a magnifying glass and fixed as described below. At least 30 ovules for each stage were used. All specimens were analyzed at the light-optical microscope. Samples with a characteristic structure (corresponding to the examined stages of ovule development) were prepared for studies with an electron microscope.

### 4.2. Light and Transmission Electron Microscopy

Selected samples of grain were fixed using a 2.5% glutaraldehyde solution in 0.1 M Na_2_HPO_4_/K_2_HPO_4_ Sorensen buffer, pH 7.3, supplemented with sucrose (15 mg/mL), for one day at 4 °C. After washing from the fixing mixture at the same buffer, the samples were post-fixed with a 1.0% solution of osmium tetroxide (OsO_4_) for 1 h, then dehydrated in increasing concentrations of ethanol (30, 50, 70, 96 and 100%), propylene oxide and encapsulated with Epon–Araldit epoxy resin kit. For light microscopy, semi-thin sections (1–2 μm) were prepared using glass knives and an ultramicrotome LKB-III (LKB, Bromma, Sweden), placed on glass slides, then stained with 0.1% toluidine blue (Merck, Darmstadt, Germany) and embedded in epoxide resin. Samples were analyzed and photographed using an Olympus BX51 microscope (Olympus, Shinjuku, Tokyo, Japan) supplied with a Color View II camera (Soft Imaging System, Münster, Germany). For electron microscopy, samples were sectioned with a diamond knife using an ultramicrotome LKB-III (LKB, Sweden), placed on formvar-coated grids and stained with uranyl acetate and lead citrate solutions. Then, thin sections were analyzed and photographed using an electron microscope H-500 (Hitachi, Ibaraki, Japan).

### 4.3. Scanning Electron Microscopy

For scanning electron microscopy (SEM), 5 seeds from 3 ears were collected from normal-size seeds (without any deformation and damage) and fixed in 2.5% glutaraldehyde in 0.1 M Sorenson buffer, pH 7.2. After having been washed with buffer, the samples dehydrated through ethanol series (30%, 50%, 70%, 96%, 2 × 100%). Then, CO_2_ for critical-point-drying (Hitachi HCP-2 critical point dryer) was applied. Dry seed fragments were mounted on a SEM stub with carbon-conductive tabs and coated with gold and palladium using an Eiko IB-3 ion-coater (Eiko, Tokyo, Japan). Samples were observed and photographed under a JSM-6380LA SEM at 20 kV (JEOL, Tokyo, Japan).

## 5. Conclusions

The results obtained in our study indicate that, in the process of ontogenesis of the wheat kernel, all the main tissues (both maternal and daughter) grow and differentiate synchronously (coordinately) until the kernel is fully matured (filling). The synthesis and accumulation of reserve substances occurs in the cells of the endosperm and the formation of protective structures of various types occurs in the integumentary tissues of the skin. A part of maternal tissues during ontogenesis dies by the PCD pathway, which is important for the normal development of wheat kernels, since this process creates space for the growth of the embryo sac and for the free (independent) growth of some integumentary layers. At the same time, further differentiation of the exocarp, endocarp (cross and tube cells) and inner integument of the ovule in wheat kernel leads to the formation of functionally different layers of the skin, which provide the embryo and endosperm with comprehensive protection, both during development and after the kernel reaches full maturity. Based on the results obtained, we claim that the formation of these tissues occurs by the path of specialized terminal differentiation, during which cells of a specific morphology are formed to perform certain functions without undergoing elimination. However, since the definition of "terminal differentiation" to characterize the life cycle of plant cells (as opposed to animals) is used quite rarely, this issue suggests further research. Investigation of the development and functioning of protective integumentary tissues in cereal plants can be quite useful for improving the storage conditions of grain in the agro-industry and long-term storage of seed collections.

## Data Availability

Data are contained within the article or Supplementary Materials.

## Supplementary Materials

The following are available online at https://www.mdpi.com/article/10.3390/plants10112538/s1, Figure S1: Fragments of exocarp cells at the early and late stages of development of wheat kernels, Figure S2: Cross (chlorophyll-bearing) cells of wheat kernel at the milk (a,b) and soft dough (c,d) stages of development (tangential sections).
