# Supplementary material for: Morphological and Ultrastructural Features of Formation of the Skin of Wheat (Triticum aestivum L.) Kernel
Source: Plants (Basel). 2021 Nov 21;10(11):2538. doi: 10.3390/plants10112538 (PMC8624426; doi:10.3390/plants10112538)
Supplement: Supplementary file 1 [file plants-10-02538-s001.zip › plants-1445015-supplementary.pdf]

Supplementary Materials

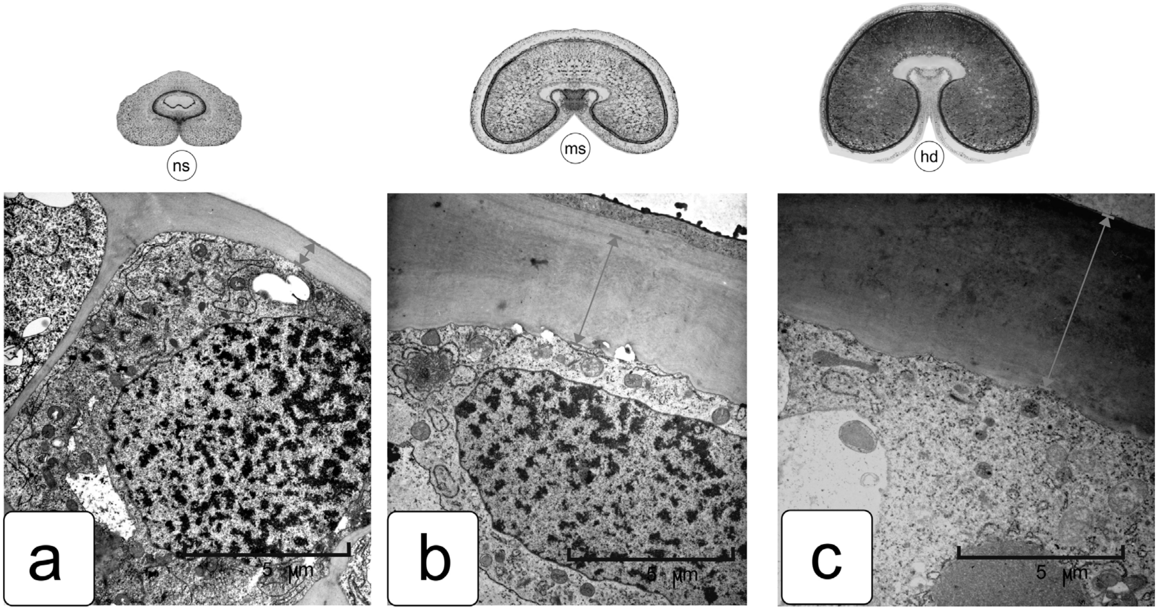

Figure S1. Fragments of exocarp cells at the early and late stages of development of wheat kernels. The degree of thickening in the cell wall to the stage of grain filling is shown. a – stage of nucleary endosperm; b – milk stage; c – soft dough stage of kernel maturation. Arrows indicate the thickness of the cell wall.

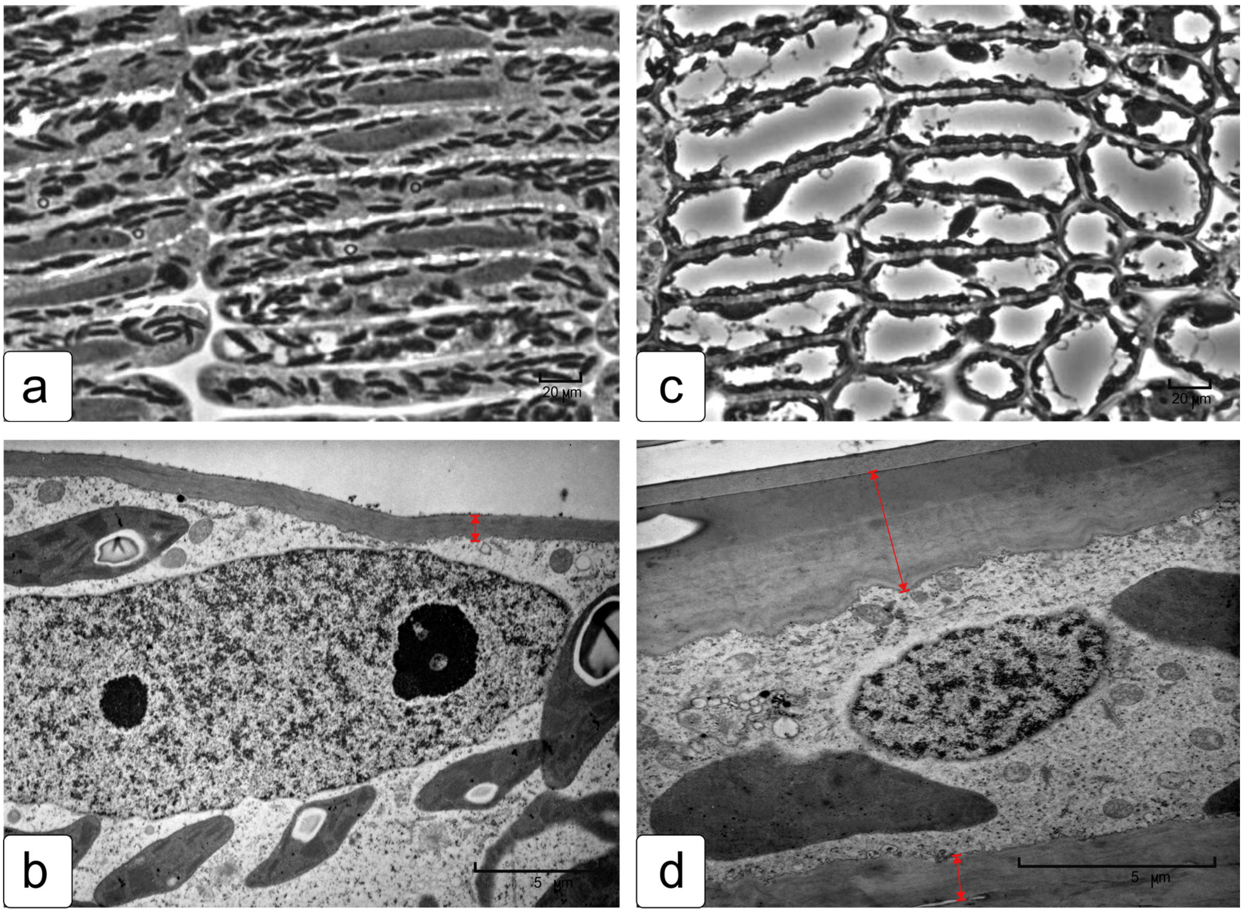

Figure S2. Cross (chlorophyll-bearing) cells of wheat kernel at the milk (a,b) and soft dough (c,d) stages of development (tangential sections). Fragments of these cells at the ultrastructure level (b, d). By the stage of grain filling, the thickness of the cell walls noticeably increases and they acquire a spiral shape (Figure 11c,d). Arrows indicate the thickness of the cell wall.
